# Anti-HMGCR (Hydroxy-3-Methylglutaryl-CoA Reductase) Myopathy: A Rare Cause of Proximal Muscle Weakness

**DOI:** 10.7759/cureus.61094

**Published:** 2024-05-26

**Authors:** Nicolas S Fink, Layla Abubshait, Amanda Deshisky

**Affiliations:** 1 Emergency Medicine, Jefferson Einstein Montgomery Hospital, East Norriton, USA

**Keywords:** hmgcr, statin, autoimmune, proximal muscle weakness, myopathy, 3-hydroxy-3-methylglutaryl-coa reductase

## Abstract

Idiopathic inflammatory myopathy (IIM) represents a rare group of autoimmune conditions resulting in muscle weakness and includes polymyositis, dermatomyositis, immune-mediated necrotizing myopathy (IMNM), overlap myositis, and inclusion body myositis. Anti-3-hydroxy-3-methylglutaryl-CoA reductase (HMGCR) antibody IMNM represents a rare but increasingly recognized subtype of IIM. Here we report a case of a 65-year-old woman on rosuvastatin who presented with two months of progressive proximal muscle weakness, significant truncal weakness, and elevated creatine kinase concerning for rhabdomyolysis and inflammatory myopathy. The patient was eventually diagnosed on day 8 of her hospital stay with anti-HMGCR antibody IMNM after delayed testing for this specific myopathy. Increased awareness of this IIM subtype, as well as its risk factors and presenting features, might improve rapidity of testing and shorten hospital stays if the diagnosis is considered in the emergency department or early in the hospital course.

## Introduction

Generalized weakness is a frequent chief complaint in the emergency department (ED) with a vast differential diagnosis and is common among elderly patients. In 2021, there were approximately 893,000 patients aged 65 years and older who presented to the ED with generalized weakness [1]. While critical diagnoses warrant the most attention, there are other significant diagnoses, such as inflammatory myopathy, which must also be considered. There has been increasing recognition of statin-associated myopathy, principally immune-mediated necrotizing myopathy (IMNM); however, it is still a relatively new entity and may not be initially considered in the differential for diffuse weakness [2,3]. The myalgia and myositis often associated with statin use have many proposed mechanisms including the inhibition of the production of CoQ 10, leading to mitochondrial dysfunction, increase in interstitial glutamate, sarcoplasmic reticulum calcium leak, and upregulation of genes involved in protein catabolism and the ubiquitin-proteosome system [4-7]. While these adverse effects of statin use are unfortunate, they are often reversible upon discontinuation of the statin medication. This contrasts with the IMNM associated with statin use, which persists even after statin discontinuation. While there has been no study to definitively demonstrate causality, statin medications are implicated in the production of anti-3-hydroxy-3-methylglutaryl-CoA reductase (HMGCR) antibodies, which, in addition to anti-signal recognition particle (SRP) antibodies, are associated with IMNM [2,3,8].

We present a case demonstrative of the continued lack of awareness of anti-HMGCR IMNM, as the patient's older age and statin use should have prompted its early consideration. Physicians should be aware of the association with statin exposure and clinical presentation of this subtype of myopathy to improve the rapidity of work-up and initiation of appropriate immunosuppressive therapy once admitted to the hospital.

## Case presentation

A 65-year-old woman with a history of type 2 diabetes, hyperlipidemia, sciatica, and endometrial cancer presented to the ED at the recommendation of their primary care doctor for an elevated creatine kinase (CK). The patient stated that approximately two months ago, she felt a sudden shooting pain down the left arm, which was short in duration and resolved over the course of the day. Approximately three days following this episode of pain, she described a sensation of heaviness and weakness in the same left arm. These sensations persisted and waxed and waned for almost one month. During that time, she also developed progressive difficulty with walking up stairs and performing activities of daily living (ADL) such as washing her hair in the shower. She ultimately recruited the help of a family member to assist her with ADLs. The patient’s primary care physician ordered a CK and informed the patient to present to the ED due to concerns for rhabdomyolysis in the setting of an elevated CK to approximately 11,000. The patient was also told to discontinue their rosuvastatin, which they had been taking for four years.

On presentation to the ED, the patient specifically denied myalgia, diplopia, dysarthria, dysphagia, dyspnea, nausea, vomiting, abdominal pain, decreased dexterity or weakness in the hands or feet, or any loss of sensation. She also denied any recent illnesses within the last two months and denied any fever, chills, or cough at the time of presentation. Initial vitals were unremarkable, with a blood pressure of 131/77, heart rate of 97 beats/minute, respiratory rate of 18 breaths/minute, SpO_2_ of 99%, and oral temperature of 36.5°C. Skin examination was significant for the absence of rash; specifically, there was no heliotrope rash, shawl sign, V sign, holster sign, or Gottron's papules. Significant positive findings on the neurologic examination revealed bilateral shoulder abduction strength of 2/5, hip flexion strength of 4/5, abdominal flexion strength of 2/5, and neck flexion strength of 2/5. Notably, the strength of all distal extremities was 5/5, with intact sensation to light touch and reflexes throughout.

Initial laboratory studies were notable for an elevated CK, transaminitis, and elevated creatinine for which one liter of lactated ringers was administered (Table 1). The leading diagnosis at that time was inflammatory myopathy, including polymyositis and inclusion body myositis. The remaining differential included rhabdomyolysis, self-limited toxic statin myopathy, and neuromuscular junction disorder. Following admission to the medicine floor, a myositis panel and antinuclear antibody were ordered, which were pan-negative (Table 2).

**Table 1:** Laboratory values upon presentation to the emergency department WBC, white blood cells; ANC, absolute neutrophil count; BUN, blood urea nitrogen; ALT, alanine aminotransferase; AST, aspartate aminotransferase; CK, creatine kinase; CRP, C-reactive protein

Component	Result	Reference Range
WBC (10^3^ cells/uL)	11.6	4.0-11.0
ANC (10^3^ cell/uL)	10.2	1.7-8.4
Creatinine (mg/dL)	1.37	0.6-1.0
BUN (mg/dL)	28	10-20
ALT (IU/L)	244	0-55
AST (IU/L)	344	5-34
CK (IU/L)	13,204	29-168
CRP (mg/L)	42.6	0-5

**Table 2:** Myositis panel and ANA results Jo-1 Ab, histidyl tRNA synthetase antibody; PL-7 Ab, anti-threonyl-tRNA synthetase antibody; PL-12 Ab, alanyl-tRNA synthetase antibody; EJ Ab, glycyl-transfer ribonucleic acid synthetase antibody; OJ Ab, isoleucyl-tRNA synthetase antibody; SRP Ab, signal recognition particle antibody; Mi2 alpha Ab, antibody against Mi2-alpha subunit of the nucleosome remodeling deacetylase (NURD) complex; Mi2 beta Ab, antibody against Mi2-beta subunit of the nucleosome remodeling deacetylase (NURD) complex; TIF-1y Ab, anti-transcriptional intermediary factor 1y antibody; NXP-2 Ab, nuclear matrix protein-2 antibody; ANA, antinuclear antibody

Test	Result	Reference Range
Jo-1 Ab	Negative	Negative
PL-7 Ab	Negative	Negative
PL-12 Ab	Negative	Negative
EJ Ab	Negative	Negative
OJ Ab	Negative	Negative
SRP Ab	Negative	Negative
Mi-2 alpha Ab	Negative	Negative
Mi-2 beta Ab	Negative	Negative
MDA-5 Ab	Negative	Negative
TIF-1y Ab	Negative	Negative
NXP-2 Ab	Negative	Negative
ANA	Negative	Negative

**Figure 1:**
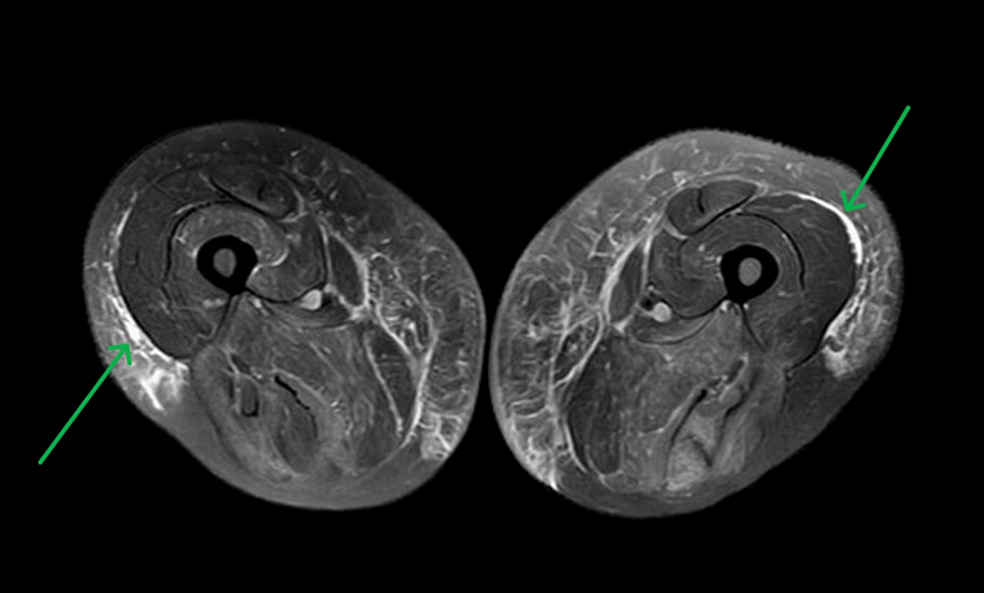
Axial T2-weighted MRI of the proximal lower extremities demonstrating hyperintensities involving all muscle compartments indicative of non-specific edema.

MRI of the lower extremities revealed interfascial edema concerning for an ongoing inflammatory process (Figure 1). The patient continued to receive maintenance fluids while on the floor for acute kidney injury (AKI) and persistently elevated CK; however, the proximal muscle weakness worsened over the course of the stay, and the patient developed diffuse non-pitting edema and pulmonary congestion due to volume overload. Fluids were discontinued, and the patient was successfully diuresed. Rheumatology was consulted, and they recommended testing for anti-HMGCR antibody, which resulted on day 8 as positive.

Following a positive HMGCR antibody, a diagnosis of IMNM was made. The patient was started on high-dose prednisone 80 mg daily; however, given the presence of edema and hyperglycemia, the decision was made to start intravenous immunoglobulin (IVIG) for two days while inpatient. The patient was discharged with rheumatology follow-up with a plan to taper prednisone dose to 60 mg daily and consider steroid-sparing therapy in the outpatient setting. Additionally, IVIG treatment would be administered once monthly. On the day of discharge, the patient had improvement in proximal muscle weakness and downtrending CK; however, she required admission to a skilled nursing facility due to persistent weakness.

## Discussion

Anti-HMGCR IMNM is one of the rarest forms of inflammatory myopathy, and its existence as a distinct clinical entity was discovered as recently as 2010 via immunoprecipitation studies [2,3]. The estimated incidence of anti-HMGCR antibodies ranges from 1.7 to 10.3 cases per million people per year depending on geographic location, and approximately 6% to 12% of inflammatory myopathy cases from longitudinal and retrospective cohort studies are anti-HMGCR antibody positive [3,8-10]. Other studies have been cited with higher proportions of anti-HMGCR positive cases; however, these studies have cohorts that consist of cases already suspected of necrotizing myopathy rather than all subtypes of inflammatory myopathy or do not clearly specify how their study cohort was constructed [11,12].

While anti-HMGCR IMNM is associated with statin use, it is unknown whether there is a direct causal link. Between 44% and 66.6% of patients with anti-HMGCR antibodies have a history of statin exposure, with the lower percentage being from a study with a relatively younger cohort [2,7]. While there appears to be an age-related factor, the rate of statin exposure with age-matched controls in other autoimmune myopathies only ranges from 25% to 33% [2]. Statins have definitively been shown to increase the expression of HMGCR, which has been postulated to result in an autoimmune response and generation of autoantibodies [13]. This provides a reasonable mechanism for the development of autoimmunity from statin exposure, and it may imply that the remaining cases of anti-HMGCR antibody IMNM develop from an upregulation of HMGCR due to exposure to a non-statin agent. The proposed mechanism for pathogenicity of these autoantibodies is ectopic expression of HMGCR or SRP on the surface of myofibers, resulting in antibody binding and complement activation [14]. There is evidence to suggest that this ectopic expression occurs during the regenerative phase of muscle cells in response to damage, such as from the myotoxicity of statins [14]. Additionally, this ectopic expression may then induce immune response in those genetically predisposed via expression of certain DRB1 alleles and subsequent presentation of antigen via HLA-DR molecules and generation of autoantibodies [14].

Overall, the differential diagnosis for diffuse weakness is quite broad and requires a detailed history, focusing on both the temporal aspects of symptoms and location of muscle weakness (upper or lower extremities, bilateral or unilateral, distal or proximal, presence of bulbar symptoms, etc.). Additionally, symptoms that may indicate neuropathy, such as sensory loss, fasciculations, cranial nerve deficits, or dysautonomia, should be assessed. The first symptoms described by our patient were most consistent with a unilateral radicular pain, and even the presence of unilateral weakness seems to be consistent with radiculopathy, plexopathy, ischemia, or neuromuscular disorder. Of course, the patient went on to develop progressive proximal muscle weakness, which is most consistent with myopathy, neuromuscular disease, or acute or chronic inflammatory demyelinating polyneuropathy (i.e., AIDP or CIDP). In particular, our patient developed significant truncal weakness, which has previously been acknowledged as a feature of IMNM [15]. It is critically important to assess the acuity of symptoms and the presence of alarm features for stroke or characteristics concerning for myasthenia gravis, such as shortness of breath, diplopia, and dysphagia. Beyond this, one might test for fatigability on physical examination or further interrogate the severity of symptoms at the beginning versus the end of the day.

While rhabdomyolysis can certainly cause proximal muscle weakness, it is an acute entity often with an identifiable cause (e.g., trauma, sympathomimetic use, exercise, statins, colchicine, ethanol, seizure, or excessive downtime) [16]. The progressive, subacute nature of the patient’s presentation argues against acute rhabdomyolysis; however, severe inflammatory myopathy may still cause acute tubular necrosis secondary to myoglobinuria and may still progress to or present as rhabdomyolysis [16-18]. Fluid administration in the setting of AKI secondary to myopathy may thus be reasonable; however, volume overload should be avoided.

## Conclusions

While the initial findings of our case are consistent with rhabdomyolysis in the setting of elevated CK and AKI, it is critically important to investigate the possible non-traumatic etiologies. Autoimmune myopathy, metabolic and endocrine derangements, and infections are a few categories that could explain the subacute presentation in this case. Additionally, self-limited toxic statin myopathy can cause proximal muscle weakness and an elevated CK level; however, symptoms resolve after cessation of the statin medication. Given that diagnoses or differentials made in the ED often determine the course and initial work-up of patients admitted to the medicine floor, it is important to consider more uncommon diagnoses when suspicion is high enough. In our case, anti-HMGCR antibodies were not tested on admission and delayed appropriate treatment, which might have shortened the patient’s hospital stay. Given that the majority of cases of anti-HMGCR antibody IMNM occur with statin exposure, the clinical presentation concerning for inflammatory myopathy with prior rosuvastatin use should have prompted an emphasis on the possibility of anti-HMGCR antibody IMNM on admission. Additionally, if testing for anti-HMGCR antibodies is not available at one's institution, recognizing these risk factors and persistence of weakness and elevated CK should prompt consideration for empiric therapy with corticosteroids.
